# A phenomenological analysis of the spiritual development of psychotherapists who work with sexual violence survivors

**DOI:** 10.3389/fpsyg.2025.1546444

**Published:** 2025-08-14

**Authors:** J. Mitchell Waters, Abigail Holland Conley

**Affiliations:** ^1^School of Mental Health Counseling, Belmont University, Nashville, TN, United States; ^2^Department of Counseling and Special Education, Virginia Commonwealth University, Richmond, VA, United States

**Keywords:** counseling, faith development, qualitative, phenomenology, trauma, religion, spirituality, sexual violence

## Abstract

Religion and spirituality play a large role in the lives of many counselors who work with survivors of sexual violence. Indeed, engagement in this clinical work may play a role in the counselor's spiritual development. In this phenomenological study, we examined the faith development experiences of religiously and spiritually diverse counselors and psychotherapists who work with survivors of sexual trauma. Through semi-structured interviews with eleven participants, the researchers identified two main themes: (1) transformative faith experiences and (2) religion as a barrier. The findings of this study highlight the intricate relationship between spirituality and faith development that counselors navigate when engaged in the challenging work of supporting survivors of sexual violence over extended periods.

## Introduction

The field of mental health counseling is a diverse profession that places a strong emphasis on fostering personal and professional development across many of its domains including clinical practice (Wong et al., 2020), supervision (Stoltenberg et al., 2014), pedagogy, social justice advocacy (Ratts et al., 2016), leadership (Meany-Walen et al., 2013), and research (Limberg et al., 2020). Development occurs across all levels of the counseling profession—counselor educators and supervisors, counseling students, clinical practitioners, and clients. In mental health professions, the significance of both personal and professional development is paramount, leading to widespread research and education on developmental theories (Benner and Mistry, 2020; Plagerson, 2015), including spiritual development.

Religion and spirituality play significant roles in the lives of many individuals in the United States (Pew Research Center, 2019) and around the world (International Center for Religion and Diplomacy, 2019), thus a key focus of this study is the domain of spiritual and faith development. While spirituality and religion might be related in some ways, they are distinct concepts (Zinnbauer et al., 1999). Religion is the practice of spirituality and involves a social context within which beliefs and practices occur (Cashwell and Young, 2011). It is institutional, historical, and creedal. Some examples are Islam, Christianity, Judaism, Taoism, or Hinduism. Spirituality, on the other hand, is “the universal human capacity to experience self-transcendence and awareness of sacred immanence, with resulting increases in self-other compassion and love” (Cashwell and Young, 2011, p. 12). Spirituality differs from religion because one does not need to practice a religion to practice spirituality as it is not necessarily rooted in the belief in a Higher Power, although it can be.

Notably, over half of the US population identifies as religious while one-fifth of the public and one-third of adults under thirty identify themselves spiritual but not religious (Pew Research Center, 2019). Given the importance of spiritual and religious beliefs and the diversity they encompass, it is important to explore the spiritual development of counselors from various spiritual backgrounds. There are numerous ways to understand spiritual development, such as attachment (Granqvist and Dickie, 2006) or relational-cultural developmental perspectives (Augustyn et al., 2017). However, Fowler and Dell's (2006) theory of faith development is the most comprehensive for the purposes of this study.

While there is a strong focus on personal and professional development within mental health professions, a major modern challenge to occupational longevity is practitioner burnout (Chang and Shin, 2021; Leung et al., 2023; Yang and Hayes, 2020) and vicarious traumatization (Ford and Courtois, 2020; Hernandez-Wolfe et al., 2015). Trauma is a common experience (World Health Organization, 2024), and many mental health professionals will encounter clients who have experienced trauma (such as sexual or intimate partner violence) whether they specialize in trauma counseling or not. Counselors who work with traumatized populations are at a higher risk of experiencing secondary and vicarious traumatization (Hensel et al., 2015). Additionally, counselors who experience these symptoms are at a higher risk of leaving the profession entirely (Cook et al., 2021). Therefore, it is important to understand how working with trauma impacts faith development, as this could provide insight into risk or protective factors for burnout.

Given the fact that spiritual development can be shaped by adversity (Pargament and Exline, 2022) and that mental health counselors who work with sexual violence are an understudied group, this study will help us gain a deeper understanding of their experiences. Therefore, the purpose of this phenomenological research was to examine the lived experiences of religiously and spiritually diverse counselors and psychotherapists who work with survivors of sexual violence. The findings of this study will help inform and improve counselor burnout prevention, counselor training, supervision, and clinical practice.

### Faith development theory

Fowler and Dell's (2006) theory of faith development is a theory for understanding how the faith and spirituality of individuals develop and change across the lifespan and involves seven stages. According to the theory, the earlier stages of faith development, *Primal Faith, Intuitive-Projective Faith*, and *Mythic-Literal Faith* occur earlier in life (infancy through early childhood) and can be described as a relatively simple, uncomplicated, and mythical faith. As individuals progress through late childhood and into adolescence, they move into the *Synthetic-Conventional Faith* stage and tend to experience their faith more interpersonally, with beliefs that are strongly but tacitly held (Parker, 2010).

As individuals age and mature, they may move into the *Individuative-Reflective Faith* stage which is characterized by experiencing spiritual dissonance and questioning where one's previously valued authorities are challenged by new understandings that come with life experience. This dissonance may necessitate a strong need for reflection and spiritual reexamination. This process, which might be described as a crisis of faith, can be painful as individuals confront the task of forging an identity that enables independent judgment about the people, institutions, and worldviews that have shaped their sense of self. At this stage, one may question inherited beliefs and traditions and might even evaluate other faith traditions for what they might have to offer. In the end, a person's existing belief system may or may not be rejected, but if retained, beliefs may be transformed and engaged with more nuance. People at this stage may also begin to experience increased tolerance and openness to the traditions of others, seeing their inherent value in ways that help deepen their own. This change might even be prompted by exposure to adversity or the adversity of others and the question of theodicy (Barnes and Moodley, 2020; Fowler and Dell, 2006). Additionally, spiritual struggles may also have the power to move individuals away from an organized faith system entirely, such as in cases of religious abuse (Ellis et al., 2022; McGraw et al., 2019) and particularly among some LGBT populations (Wood and Conley, 2014).

If individuals continue in their faith development, they might experience stage five, *Conjunctive Faith*, which is characterized by greater tolerance for paradox, perspective-taking, and the desire to think beyond either/or dichotomies (Parker, 2010). While moving fully into this stage is rare, even rarer still is the final stage, or *Universalizing Faith*, which is where an individual begins to see themselves as part of a universal collective that is concerned with the whole rather than the individual. (Fowler 1995) described these individuals as possessing “a special grace that makes them seem more lucid, more simple, and yet somehow more fully human than the rest of us (p. 232).” While Faith Development Theory (Fowler and Dell, 2006) was used to guide this study, it is important to note that stage theories such as this have received criticism for reducing development to a linear fashion (Coyle, 2011; Pelaez et al., 2008). Many people may experience growth both cyclically and between stages in a way that is not adequately captured by this theory.

### Faith development in mental health practice

In his seminal article, (Pargament 2013) described spirituality as a process and a search for the sacred that evolves and develops over time. His definition of spirituality posits that humans “are motivated to discover something sacred in their lives, hold on to or conserve a relationship with the sacred, and when necessary, transform their understanding of the sacred” (p. 260). This aligns well with Fowler and Dell's (2006) theory that demonstrates the developmental nature of faith and spirituality across the lifespan. It also paves the way for a rich understanding of how faith changes can be prompted by adverse life experiences.

Prior research has shown that many turn to their religious and/or spiritual beliefs to make meaning in the face of adversity (Carroll et al., 2020; Thomas and Barbato, 2020; Voytenko et al., 2023). Additionally, research indicates that counselors who work with distressed and traumatized clients may often draw upon their religious or spiritual beliefs to find meaning in the suffering their clients have endured (Hardiman and Simmonds, 2013). Another common experience for religiously and/or spiritually affiliated counselors is for beliefs about their religion or spirituality to change over time as a result of their work (Kim and Kim, 2023).

(Martinez and Baker 2000) utilized a grounded theory analysis to examine psychodynamically-oriented religious counselors in their training, supervision, and practice in the United Kingdom. One of the most salient themes researchers identified was *changes occurring in participants' religiosity*. The study highlighted the fact that faith changes often occur in counseling practice, facilitated by exposure to the diverse experiences of diverse clients.

In another study, (Simşir et al. 2017) examined the religious and spiritual development, post-traumatic growth, and the changes that a traumatic experience causes in one's religious and spiritual life in a sample of individuals with traumatic spinal cord injuries. The themes that emerged that were related to spiritual change, growth, or development were *changes in attitudes toward God, religious practices, changes in belief in fate and an afterlife*, and *religious participation*. The findings of the study revealed that the participants experienced a great deal of post-traumatic growth following paralysis that they attributed to their faith.

Another important concept related to spiritual development and adversity is that of doubt. Doubt can serve different functions for individuals in their faith development and may result in varying emotional experiences. Doubt may lead to both spiritual growth or decline (Pargament and Exline, 2022). Spiritual struggles with doubt represent potentially significant crossroads leading to distress, disorientation, and cognitive dissonance. Whether doubt may be considered positive or negative may depend on the individual's subjective experience of the function doubt served in hindsight. That is, while doubt may be an inherently uncomfortable experience in the moment, doubt may considered a more negative or positive experience if it ultimately led the individual to places of further disillusionment and discomfort or to places of new spiritual possibilities.

It is clear that religious and/or spiritual individuals who encounter adversity, even indirectly, may experience changes in their religious and/or spiritual beliefs (Pargament and Exline, 2022). Additionally, religion can also be a source of stress, adversity, or trauma at times (Ellis et al., 2022). The function that religion and spirituality might play in the lives of individuals who experience adversity is complex and multilayered. Very few studies have examined the spiritual development of counselors generally or, more specifically, how experiences of adversity may impact their spiritual development. In line with Fowler and Dell's (2006) theory of faith development, the interactions counselors have with their clients and the ways they impact them may serve as a catalyst for the counselor's own spiritual development.

While few studies have examined the spiritual development of counselors and other studies that have examined the spiritual development of individuals that have experienced adversity (Daniel, 2017; Leo et al., 2021; Van Deusen and Courtois, 2015), there are currently no studies on the spiritual development of counselors who work with clients that have experienced significant adversity. Globally, one in three women will have experienced sexual or intimate partner violence in their lifetime (World Health Organization, 2021). Therefore, counselors have a high likelihood of working with clients who have experienced sexual trauma. Additionally, there is a paucity of literature that has examined faith development across diverse faith and spirituality beliefs in the same sample, including those who identify as spiritual but not religious. The purpose of this study, therefore, was to perform a qualitative investigation into the experiences of the faith development of religiously and spiritually diverse counselors who work with survivors of sexual trauma.

## Materials and methods

The primary research questions that guided this study were: What are the lived experiences of religiously and spiritually diverse counselors who work with survivors of sexual violence and how do these experiences impact their spiritual development? To answer this question, a transcendental phenomenological investigation was conducted. The focus of phenomenology is discovering the essence of the lived experiences of individuals and groups (Van Manen, 2014). Transcendental phenomenology is derived from the philosophical work of (Husserl 1931) and later applied as a qualitative research approach by (Moustakas 1994). It is a descriptive approach with the aim to analyze “the essences perceived by consciousness with regard to individual experiences” (Padilla-Díaz, 2015, p. 103).

This approach to qualitative inquiry was fitting for this study because the experiences of spiritual development in religiously and spiritually diverse counselors are complex and may vary from counselor to counselor. Semi-structured individual interviews were the primary data collection method, which was appropriate given the personal and sensitive nature of this study. Participants were provided with an informed consent document prior to the interview, where they were notified of the personal nature of this study. In line with IRB protocol and to increase a sense of emotional safety, participants were informed that they were free to decline to answer any questions for any reason and could discontinue the interview and participation in the study at any time and for any reason. A copy of the interview script all participants were asked can be found in Appendix A.

### Positionality

The researchers included two white counselor educators, one male identified, and one female identified, at CACREP-accredited universities. The lead researcher's interest in the topics of this study stems from his own experience. Spirituality is important to the lead researcher as an individual who identifies as a Protestant and a counselor who works in the treatment of traumatic stress from sexual violence. The second author has a personal and professional interest in spirituality and working with survivors of sexual violence. Both researchers also have experienced vicarious traumatization and have been aware of ways their spirituality intersects with it.

Throughout this research process, there were some assumptions the researchers kept in mind and constantly re-evaluated. One such assumption is that there are many variations of religious and spiritual identities and attitudes, and not everyone will experience these issues the same way. Another assumption that was bracketed was that just because a counselor is religious and/or spiritual does not necessarily mean that they engage with their religion or spirituality to cope with work-related stress.

### Participants and procedures

Upon obtaining approval by the institutional review board, recruitment emails were sent out through counseling listservs such as CESNET and ASERVIC Connect, directly to counseling program coordinators and university counseling centers to forward to program alumni and individual practitioners, and through online public counseling directories such as Psychology Today and South Asian Therapists. After an amendment, approval was granted to recruit international participants for this study to gain a more diverse, representative sample. If potential participants expressed interest in participating in the study, they received a Google Forms link where they were directed to provide the basic demographic information of their name, gender, race, age, and religious and/or spiritual affiliation.

For participants to be considered for inclusion for this study, they had to meet the following criteria. Participants had to: (1) be at least 21 years of age; (2) speak English, (3) have access to the internet, camera, and a microphone; (4) have a minimum of a Master's degree in counseling or a closely related field; (5) have been an actively practicing counselor who specializes in working with survivors of sexual violence for at least 2 years; and (6) have a religious and/or spiritual identity that is integral to their personhood. The 2-year requirement was implemented because faith changes as a result of working with trauma may develop over time. The participants chosen based on the criteria were interviewed over Zoom and were contacted again during the data analysis phase for member checking. Interview questions were crafted with the aim to examine the participants' experiences. To help with this process, before the interviews took place, a pilot test was conducted to test the protocol questions with a counselor who met all the inclusion criteria but was not included in the final sample for this study. The feedback from the individual was that the flow and structure of the questions helped elicit rich data.

The participants came from diverse work settings including military bases, private practice, chemical dependency treatment facilities, community mental health clinics, religious centers, universities, and schools. The participants were assigned pseudonyms for the protection of their identity and were selected to accurately reflect the cultural backgrounds of the participants. Individuals with the same religious, racial, and/or cultural identities of the participants were consulted for pseudonym selection to ensure accuracy. Participants self-identified for every demographic question. Participant demographics can be found in Table 1.

**Table 1:** Participant demographics.

**Pseudonym**	**Religious and/or spiritual identity**	**Race/ethnicity**	**Gender**	**Country**
Jackie	Christian	White	Female	USA
Kathy	Non-Religious; Spiritual (Collective Consciousness)	White	Female	USA
Eveline	Christian	White	Female	USA
Adam	Christian	Indian	Male	USA
Aisha	Muslim	Pakistani British	Female	UK
Abdul	Muslim	South Asian American	Male	USA
Myra	Christian	Indian	Female	India
Isabel	Christian, Wicca/Celtic Shamanism	Puerto Rican/American	Female	USA
Giti	Hindu	South Asian	Female	Canada
Fatima	Muslim	Middle Eastern American	Female	USA
Anima	Muslim	South Asian American	Female	USA

### Data analysis

The phenomenological data analysis (Moustakas, 1994) followed a series of steps: (1) epoche; (2) phenomenological reduction; (3) imaginative variation; and (4) synthesis. In the first stage, epoche, or “to refrain from judgment” (Moustakas, 1994, p. 33), we set aside, or bracketed, everyday assumptions and biases as the phenomenon was explored and engaged in reflexive journaling. While achieving complete objectivity in qualitative research is not feasible, this process helped mitigate potential biases that could have influenced the findings.

After seeing aside our preconceived beliefs and assumptions, we were able to engage in the second step, phenomenological reduction, and focus solely on how the participants' experienced the phenomenon (Merriam and Tisdell, 2016). By continually returning to their descriptions and intentionally bracketing external interpretations, the phenomenon was isolated and examined in its pure form, allowing for a deeper understanding of its underlying structure and meaning. To achieve this, we utilized horizontalization, or the process of laying out all the data for examination and treating each piece as equally significant (Moustakas, 1994). We then distilled these horizon statements into non-repetitive, non-overlapping statements, which were grouped into meaning units representing initial themes and codes. Individual textual descriptions were then formed that describe the invariant meaning units.

The third stage, imaginative variation, entailed generating possible meanings for textural themes identified in earlier steps, and considering them from various perspectives. Two external auditors reviewed the transcripts and coded and provided feedback for refining the codebook. After incorporating their insights, we identified structural themes reflecting how the participants experienced the phenomenon. Finally, we synthesized the textual and structural themes for all participants to obtain the essence of their experiences (Moustakas, 1994). At this stage, all codes and themes were finalized, and the essences were thoughtfully organized to create essential themes that concisely explained the data and were reflective of the participants' experiences. Implications were then derived by examining how the themes revealed participant needs and areas for growth in the profession. These, then, informed recommendations for practice and counselor preparation.

### Trustworthiness

To maximize trustworthiness, there were several steps taken in this study. Firstly, assumptions were bracketed, and judgments were suspended to gain a more accurate understanding of participants' experiences. Secondly, reflexive journaling was done where thoughts and feelings we experienced during data collection were written regarding potential bias that could have tainted the results. Thirdly, member checking and coding calibration was also completed. Member checking allowed for participants to clarify or correct initial interpretations of the data. Four of the participants responded to the member-checking prompt and provided their feedback. Two outside coders were then brought in to analyze two separate transcripts and provide their feedback. The updated codebook was then audited by a counselor educator and an educational psychologist. The codebook was revised again implementing their feedback. A final round of coding for all transcripts was done using the final version of the codebook and developed the themes and subthemes. The themes and subthemes were developed based on the number of participants who made statements consistent with a theme and when certain codes aligned together frequently.

## Results

Two major themes and four subthemes (see Table 2) emerged that were representative of the participants' experiences or faith development: *Transformative Faith Experiences* and *Religion as a Barrier*.

**Table 2:** Themes.

**Themes**	**Subthemes**
Transformative faith experiences	a) Changes in belief
	b) Changes in practice
Religion as a barrier	a) Resistance to pressured forgiveness
	b) Religious institutions as a barrier to healing

### Theme 1: transformative faith experiences

The first major theme, *Transformative Faith Experiences*, can be defined as changes in religious and/or spiritual beliefs as a result of their work with survivors of sexual violence. The participants indicated that they could not enter therapeutic relationships with these survivors and come away unchanged. As a result of their work and continuous exposure to clients' trauma narratives, the faith changes experienced by the participants were arranged into two subthemes: (a) changes in their beliefs and (b) changes in their practices. Changes in faith beliefs, for the purpose of this study, are described as changes in views or conceptions of God or their Higher Power, whereas changes in faith practices indicate changes in specific rituals or modes of interacting with God or their Higher Power, such as praying, journaling, or meditating.

#### Changes in belief

The participants experienced significant changes in their beliefs as a result of their work with survivors. Changes in participants' beliefs may be described as positive and negative, according to criteria set forth by religious coping (Pargament et al., 2011) and spiritual coping (Charzyńska, 2015) scales. Positive religious and positive spiritual coping are types of coping that reflect secure attachment with a Higher Power or nature, spiritual connectedness with others, and a more positive worldview. It is reflected through an individual's behavior, thoughts, and attitudes during stress and is typically associated with more positive psychological outcomes following stress or adversity.

Negative religious and negative spiritual coping conversely involve behaviors, thoughts, and attitudes that facilitate spiritual tension, conflict and struggle with a Higher Power or nature and others, negative reappraisals of one's Higher Power's power or the benevolence of the universe, spiritual questioning, and interpersonal discontent (Charzyńska, 2015; Pargament et al., 2011). Negative religious and spiritual coping is typically associated with poorer psychological and relational outcomes following stress or adversity.

The majority of the participants felt that their beliefs changed positively, such as experiencing a deeper reliance on God, increased intimacy with Him, or discovering different aspects of God's character. When asked about changes in his faith, Abdul stated, “I think it has deepened my connection with God, spiritual connection, and appreciating different views and beliefs that exist in the religion.” Jackie shared a similar experience as she stated:

I don't find myself being shaky [in my faith] because I have had the privilege of watching God do so many beautiful things in so many different people's lives; from the age of two all the way up, having experienced a vast array of really traumatic experiences, from having their loved ones killed in front of them at a mass shooting, at church of all places, to incest for the little kid, or being raped…all kinds of situations. So, my faith is not as egocentric as it once were.

Abdul and Jackie felt that their work with survivors had increased their appreciation of God and nuanced their understanding of God, despite the horrific stories they have heard. That is, the participants had experienced changes in both their intellectual understanding of and emotional relation with God. The participants experienced changes on both cognitive and relational levels because of their work with survivors, which are related in some ways but represent different modes of spiritual experience.

The participants also felt that their work with survivors deepened their understanding or conceptualization of God and theology. Aisha stated, “Ya Shafi is a name of God that means ‘The Healer.' I find myself, as I'm doing the work, I'm reciting the name Ya Shafi… I knew that there were 99 names for Allah. I knew they're important, but I didn't realize how important until these latter years of my life.” Aisha felt that her work helped her relate to Allah more through the name “Ya Shafi” because she also identifies as a healer.

Adam stated that his spiritual beliefs had not changed as a result of his work, but strengthened. He stated, “My spiritual life has not changed. It has strengthened because I have seen a lot of people out in the world who are suffering, who need help… If you want to use the word, ‘changed' maybe so, but my spiritual life… that didn't change.” Adam shared that, with all the other changes he has experienced in his work, he felt that his spiritual life was one of the few constants. Similarly, Isabel explained that her religious identity and perception of religious possibilities changed substantially because of her interactions with her clients. She stated:

One of the ways that things have opened up for me is by meeting survivors who lost their original religious tradition. And they were exploring things like Reiki or Shamanism or Wicca or paganism. I learned about more diverse, spiritual paths from clients that were survivors because… the church had let them down by not listening to them… So, I learned about different religious paths outside of like the mainstream ones, really from my clients. And it opened up my eyes to a lot of different spiritual practices that really resonate with me as a person.

Indeed, Isabel's religious identity changed because of her survivor clients' religious journeys and the harm experienced from the church. Integrating Wicca into her Christian identity was also a process that became further solidified from her interactions with her survivor clients.

Although there were positive changes in faith beliefs as a result of their work with survivors, some participants also experienced negative changes in their beliefs. This is not an uncommon phenomenon given what is known about how consistent exposure to clients' traumas may impact beliefs and worldviews negatively. However, these changes should also be examined with nuance because negative and positive changes in spirituality can co-exist. Giti demonstrated this idea when she stated:

I have to be quite honest with you. Sometimes I question God's existence because, how can something so traumatic happened to these innocent people? But I think what brings me back to not being in that angry state is that there's a trust. There's a trust that this person is a survivor and is here for a reason and is here to realize their potential. I don't believe that anything happens just by coincidence. I think God has a plan and this is this person's journey to get to where they need to be.

Giti was dealing with the question of theodicy. Because she had seen so much trauma and what one might consider “evil,” she began to question God's existence in the first place. Even as she questioned God's existence, Giti also expressed her firm belief that God has a plan for everyone. To further illustrate the complexities in faith changes, Anima similarly stated:

The pillars, the things that are said. You're supposed to believe in God, you're supposed to believe that Mohammed is the last prophet. That is not wavering. But the explanation that I've been given is that your reason for living is to worship, which yes, fine. But God doesn't need that so there has to be… something bigger that we just can't understand or has been missing from my culture's definition of Islam.

Anima felt that her work with survivors had exposed some of her unanswered questions or incomplete understandings of the purpose of existence and Islam. While most of the participants felt that their beliefs changed positively because of their work with survivors, some participants felt that their work had led to doubt and questioning. Whether the participants experienced more positive or negative changes in their beliefs, what remained consistent across the participants was the fact that they could not engage in this work with survivors and be exposed to such traumatic stories and not walk away unchanged.

#### Changes in practice

Faith changes also occurred in the ways that that many participants practiced. Common religious/spiritual practices include prayer, meditation, journaling, attending religious services, practicing gratitude, and spending time connecting with nature. Eveline reflected on ways that her spiritual practices had changed because of her work with survivors and stated, “I have to take care of my prayer time. Prayer time is not just ‘pray and I'm done' or ‘read the Bible and I'm done.' It's about dedicating my body to the Lord too.” Eveline's experiences with sexual trauma survivors helped her grow in understanding about how sacred the body is and that it should be dedicated to God.

Fatima also felt that she changed the ways that she prays because of her work, she stated “In the Islamic tradition, there's the obligation to the five ritual prayers. But then there's prayer of just talking to God…I feel like I've been more personalizing my prayers in that way and addressing certain nuances.” Fatima felt that her work with survivors has prompted deeper intimacy in the ways she prays and a more personable relationship with God. Anima had similar experiences in her engagement with Islamic prayer. She stated:

I find myself trying to connect more spiritually with my belief system. In Islam, you're supposed to pray five times a day and all that fun stuff but sometimes, it's hard to do that realistically. I think, because of that, my focus has shifted from the “rituals” that I'm supposed to do, but I make sure that I feel spiritually connected.

This quote is complex, however, because it is followed with Anima also stating, “But then it also like makes it harder to put myself in in religious situations or environments that contribute to the quote-unquote ‘problem.” This nuance highlights the complexity of the participant's experience because she finds it important to be “spiritually connected,” yet also understands how some religious institutions can create the conditions for the perpetration of sexual violence. The participants' experiences with this phenomenon will be detailed in the next theme.

Aisha described feeling like she has more of an “attitude of gratitude” now. This attitude has contributed to how she found herself more consistently praying and how she had “begun to become further in touch with the scriptures and history” and “doing further research about women and their rights in Islam.” Aisha summarized her experiences by stating, “Although I say the term practicing, I integrate the practice more than I perhaps did before.”

Fatima, Anima, and Aisha, who all identified as Muslim, all experienced this change in the ways that they pray and experience Allah and felt that they can approach Him in a more personal way. These changes in spiritual practices have created a deeper sense of connection and unity with Allah.

Overall, most of the participants experienced either changes in their faith in terms of their belief, practices, or both. Some participants experienced positive changes in the ways they conceptualize God while others experienced heightened doubt and uncertainty. These changes were highly unique to the individual participants as evidenced by the fact that not all of them experienced changes in the same ways. While all participants experienced some growth or positive development in their spirituality, there were also perceptions that religion can function as a barrier at times.

### Religion as a barrier

The second major theme, *Religion as a Barrier*, can be described as the ways that the participants perceived that institutionalized religion can function as a barrier for survivors' healing and recovery at times or even perpetuate sexual violence itself. Although many of the participants described drawing strength and hope from their religious affiliations, some of them also perceived that these institutions can be damaging to survivors as well. Two subthemes emerged within this category: (a) religious institutions as a barrier to healing and (b) resistance to pressured forgiveness.

#### Religious institutions as a barrier to healing

Some participants felt that some religious institutions can serve as a barrier for healing following sexual trauma. Kathy, a counselor that has had experiences with Catholic and Christian faiths and now identifies as spiritual but not religious, summarized this phenomenon well when she stated:

It's amazing how many times people from various religions show up, mostly Christian, which I think is really interesting, because I'm not. And I think that, having gone through my own experience with that and hearing very similar experiences, it just reaffirms for me that the dogma and doctrine of religion in the hands of humans… important point, *in the hands of humans*, becomes problematic for people who are experiencing this sort of thing because it's not helpful. But if you can set aside the dogma and doctrine, and just the spirit of the religion, or spirituality in my case, that can be an incredible asset in the healing process. Unfortunately, humans tend to make mistakes.

Kathy perceived that religious doctrine combined with the imperfection of humanity can impede a survivor's journey to healing and recovery. She further explained how religious institutions can perpetuate shame for survivors using the word “sin.” Kathy stated that “the word ‘sin' becomes very toxic, and it fractures and splinters into so many different areas of a person's recovery process… It gets really murky.” That is, Kathy perceives that the concept of “sin” in some religious institutions may impede a survivors' recovery process as elements such as shame or undue responsibility for the violence may be placed upon or internalized by the survivor.

Similarly, Isabel perceived that some religious institutions send the message that “if you are a religious person, you cannot enjoy your sexual side.” Isabel felt that some religious doctrines associate sex with shame, thus, religious survivors of sexual violence also experience this shame. She also shared a story about a survivor client who attended “a conservative mainstream kind of church,” reported the assault to their pastor, and the “pastor brought the victimizer and the survivor together to have a conversation.” This significantly emotionally impacted both Isabel and her client in negative ways. Isabel reported telling her client, “Look, I'm not in the business of telling anybody anything, but if I were you, I would not go back there.” Isabel has witnessed her survivors being harmed by the church and some of them have either changed their religious affiliations or left the institution altogether.

Some participants felt that religious institutions might contribute to the creation of the conditions for sexual violence to be perpetuated at times, even if the act is not condoned by religious communities or leaders. This was an uncomfortable topic for many of the participants because it necessitated the need to try to reconcile the fact that what they frequently turn to for meaning-making may also be part of the problem at a structural, institutional level. For example, Anima noted that there can sometimes be religious abuse and “using religion as a means of control which often leads to sexual trauma” “makes [her] very angry.” Likewise, Aisha stated that they were, “Not just Muslim clients but… I have a client who comes from a Christian background, and she'd completely veered away from Christianity…” She went on to also share that, throughout the course of treatment, that client expressed that she “realized that ‘I can still have some of those elements Christianity did to me, but I don't necessarily have to take on the abuse.” Myra had a similar perspective and stated:

With [my] patients that are Christian and they bring in their faith…. most of them are pretty anti-institution, anti-church. They're so upset with the way the institution has handled [sexuality issues] and often, the perpetrators are within the system. The very people who are supposed to mentor you, shepherd you. Whether it was your Sunday school teacher, whether it was your pastor, whether it was whoever. And then they're like, “Well, if that's what they represent, we don't want to have anything to do with God.”

It is worthy to note that Myra is a Christian residing in India, a predominantly Hindu country. While she self-identifies as Christian, she notices that many of her patients that are survivors feel angry with the church with regards to their approach to issues of sexual violence. Furthermore, her clients have been sexually assaulted by members and, at times, church leaders. Members of the institution that represents a salient identity for Myra have indeed contributed to the problem at times. This might have facilitated some intrapersonal discord within her as she stated:

I've had my own history with [trauma] and I have moved from a space of “why me?”… to a “I never leave you alone, forsake you” and all those. But where were you when this was happening?… And honestly, I don't have an answer. I'm sure He was there, but for some reason, it was allowed.

Myra's experiences of existing in and drawing strength from the same religious affiliation that she has also seen harm her survivor clients indeed created tension within her.

Fatima also shared that she sometimes felt that male religious leaders aren't sensitive to how large and serious of a problem sexual violence is. She stated:

A lot of people, women especially, feel like either Imams or scholars or men don't understand or aren't sensitive to it, simply because maybe they didn't have that space. Or, at a young age, they were shut down or made to feel ashamed. I think understanding that this is an integrated part or an intersecting part of their identity… Part of the trauma is not just the sexual trauma, but it's the trauma of feeling cut off from the community or the perception of feeling judged from the community or “I'm dirty” or “I'm bad spiritually.”

That is, Fatima felt that certain individuals in the patriarchal culture that she existed in did not lend credence to the severity of sexual violence. Felt that they even shamed survivors at times, either directly by their words or indirectly by failing to address it.

Finally, Isabel, while not specifically related to sexual violence, perceived that some religious institutions stigmatize mental illness and create barriers for clients to move toward wellness. She stated, “There were people there that would say, if you are a Schizophrenic, you should not be taking medication. You should deepen your relationship with God. And I was like, ‘You people are freaking crazy.” While Isabel strongly believes in the power of God and her faith is dear to her, she did not believe that it should override best practices.

These participants perceived that some religious institution contribute to the problem. They felt that some of these institutions associate sex with shame, thus, constructing a narrative that prevent survivors' progress toward healing and recovery. This is noteworthy because these same participants also felt that religion and/or spirituality are extremely important aspects of their identities, so understanding the intrapersonal tension or conflict that occurs is essential.

#### Resistance to pressured forgiveness

A few of the participants also reported experiencing feelings of resistance to, or anger about, religious institutions and/or leaders that pressure or coerce survivors into forgiving their attackers prematurely. Jackie stated, “If I do have someone who comes in and they're sharing how they've gone to their church, or priest, or pastor, how this idea that they have to ask for forgiveness… the narrative is so wrong on that.” When describing her approach to sexual trauma therapy, Jackie expressed wishing the “tide would turn” on the notion that there is any sin on the side of the survivor and that the survivor must forgive his or her attacker.

Isabel experienced this same resistance because of the negative impact that this religious pressure has had on her survivor clients. She will even go as far as to tell her clients that they are not obligated to forgive, even if the client is religious themselves. She stated:

When it comes to people who have survived, whether it's being molested or incest or a sexual assault, the first thing that always comes to mind that I hear from other people is, “How do you discuss forgiveness with your clients?” And the reality is, I tell my clients, “You don't have to forgive.” Even if how you were raised and somebody comes in and they're Catholic and they're like, “you know, if I don't forgive this person…” and I'm like, “You don't have to forgive anybody.” Sometimes forgiveness is actually about the other person. And we're forgiving out of obligation because the Bible tells us.

Isabel elaborated on her beliefs and experiences with forgiveness by saying, “What's the other person going to say? I'm sorry that I did this to you? That doesn't mean dick.” She also emphasized the fact that many survivors feel obligated to forgive and noted, “[they're] not obligated for shit. This person came in and took something from you. That is a gift God gave you in my opinion.” Isabel felt strongly that the survivors were not at fault for any part of their traumatic experiences and, thus, had no need to ask anyone for forgiveness.

Related to forgiveness is also the concept of repentance. That is, forgiveness is something one gives to another that has wronged them and repentance is when one experiences the feeling of sincere regret or remorse about their wrongdoing and actively seeks absolution. The participants expressed resistance to survivors feeling pressured to engage in either one of these because they felt that survivors do not need to forgive their attackers prematurely and that survivors have done nothing wrong that should warrant repentance. Fatima spoke into this as she shared about one instance in particular:

I was presenting a case around sexual trauma in a training. One of the Imams who was presenting with us, when I mentioned that this client started to engage in repentance… he immediately was like, “Make sure she's not repenting for the sexual abuse.” She was touched by that… I checked in with her and she was like, “I just find saying that word in Arabic,” but Allah, she found it comforting. But it was important to make sure that her sense of feeling dirty, that that is just his dirtiness, the abuser, being projected onto her.

Here, Fatima described a narrative contrary to the previous quotes, where a religious leader was able to assist in the healing process by also rejecting the notion that a survivor should have to repent for experiencing sexual trauma. In this instance, a religious leader served as an instrument that promoted healing and recovery for a survivor rather than acting as a barrier to it.

However, a confirmatory example arose in Adam's explanation of his clinical and spiritual approach to working with survivors. He noted, “I try to tell them, ‘I've made a lot of mistakes, but it's what I had learned from my mistakes,” suggesting that sexual violence was a mistake on the survivor's end. Adam also shared the example of “The Woman at the Well,” a Christian parable where he noted:

Jesus did not condemn her. Jesus knew exactly what she was going through. He asked a question, when she said, “I don't have any husband.” Jesus said, “That is true what you're saying. What you have is not your husband.” She knew what exactly. So… I try to cultivate the field, to make it is good for sowing the seed. So, that's one of the theories that I have learned, that in those circumstances, that's exactly the same way that Jesus had done. When the time was right, then he said that, “Go and sin no more.”

While Adam did not explicitly state that survivors should ask for forgiveness, the notion that a victim of sexual violence has committed a sin. Sin, in Christian faith, must be atoned for by asking for forgiveness and making reparations. These examples are connected to Kathy's previous quote where she expresses the idea that “religion in the hands of humans” can serve as both a resource and a barrier at times.

## Discussion

This qualitative study examined the faith and spiritual development of religiously and spiritually diverse counselors who work with survivors of sexual trauma. The participants in this study reflected five distinct religious and/or spiritual identities, came from four different countries, and identified with multiple ethnic backgrounds. It is important to reiterate that, while steps were taken to increase trustworthiness, qualitative data interpretation is always shaped by the positionality of the researchers and results may align with the researchers' own experiences as stated in the positionality section.

This study builds on prior research on faith development theory (Fowler and Dell, 2006), which posits that religious and spiritual beliefs may evolve over the lifespan. Importantly, this study supports the notion that indirect exposure to adversity may act as a catalyst for transitions between developmental stages. Previous studies have shown that individuals who experience trauma often report changes in their religious or spiritual beliefs (Krause and Hayward, 2012; Leo et al., 2021; Robinson, 2014). However, limited research has examined how vicarious trauma, particularly among counselors that work with survivors of sexual trauma, may influence such belief systems. Findings from the present study indicate that many participants experienced significant changes in their faith beliefs, suggesting a meaningful relationship between vicarious trauma and spiritual development.

In this study, many participants experienced positive changes in their religious and/or spiritual beliefs that they felt were directly attributed to their work with survivors. Five of the participants felt that their religious and/or spiritual beliefs changed for the better, as evidenced by experiencing deeper intimacy with God, discovering different aspects of His character, or experiencing increased reliance on Him. These findings align with and extend prior research suggesting that individuals who identify as religious and/or spiritual look to God or their Higher Power in times of stress and adversity. This spiritual engagement can foster comfort, meaning, relational intimacy (Pargament et al., 2011), and greater degrees of post-traumatic growth (Schaefer et al., 2018). Within the current sample, participants described similar processes, reinforcing the idea that spirituality may serve as a coping mechanism in the context of vicarious trauma. Notably, several participants also reported experiences such as questioning, doubt, and spiritual struggle, providing a key example of how adversity, even indirectly, may help promote forward motion in faith development (Fowler and Dell, 2006). Therefore, vicarious trauma may function not only as a stressor but also a potential catalyst for deeper spiritual reflection and development.

All these experiences, regardless of direction and specific religious affiliation, demonstrated the profound impact that working with survivors has on an individuals' religious and/or spiritual identity and development. Religion and spirituality can serve as central meaning-making frameworks for many individuals across cultures and contexts (Bryant-Davis et al., 2011; Dale and Daniel, 2011). Given this, it is unsurprising that continuous exposure to traumatic narratives would impact how individuals interpret their experiences and make sense of the world. For many participants, this also necessitated a corresponding change in their religious or spiritual practices, reflecting an evolving response to the demands of their trauma work.

Many participants noted an increase in spiritual and reflective practices—such as prayer, meditation, journaling, and reading sacred texts—directly influenced by their clinical work with survivors. The findings in this subtheme appeared to be closely related to, or a product of, changes in belief. For instance, because one participant had experienced increased intimacy with God because of her work, she also felt that her prayers increased both in frequency and closeness. Similarly, two other participants experienced positive changes in the ways that they engaged with and experienced Allah. That is, they began to engage with Him in a more personable way that felt more meaningful to them. These findings highlight the reciprocal relationship between belief and practice, further illustrating how vicarious trauma can shape not only internal faith orientations but also external spiritual practices.

For the majority of this sample, it appeared that providing counseling services to survivors of sexual violence over an extended period inevitably influenced their religious and/or spiritual beliefs or practices. This finding is consistent with previous literature that both direct and secondary exposure to trauma can affect meaning making systems, such as one's spirituality (McMartin and Hall, 2022). Although many of the participants felt that their religious and/or spiritual identity served as a strong protective factor that helped mitigate trauma symptoms, there were also some that perceived that religion could also function as a barrier to wellness and trauma recovery. In these cases, certain religious beliefs or community messages were perceived as hinderances to processing trauma or accessing support. These divergent experiences underscore the complex roles that spirituality and/or religion can play in coping with trauma.

Six participants perceived that religious institutions can act as a barrier to healing for survivors at times. Because of this, the participants experienced intrapersonal tension as they struggled to hold two truths simultaneously together: the deep importance of their religious beliefs and the fact that they perceive that it can impede survivors' recovery process or can even contribute to maintaining the conditions where sexual violence may be perpetuated. While negative perceptions of organized religion are not uncommon (Pew Research Center, 2019), these findings highlight the need to examine such tensions with nuance, particularly in the context of trauma-informed counseling.

Participants reported various ways in which religious messages and institutional practices contributed to survivor shame. Two noted that teachings associating sex with shame (i.e., “dirty” or “bad spiritually”), can lead their survivor clients to internalize shame with their trauma. Similarly, one participant described how some religion's fixation on “sin” also perpetuates a constant state of shame for her religious survivor clients. In more extreme cases, religion was seen as undermining mental health treatment. For instance, one participant's recalled a religious leader discouraging psychiatric medication in favor of prayer for individuals with schizophrenia, highlighting a misalignment between some religious views and counseling best practices (Grover et al., 2016).

Participants also highlighted how religions institutions have historically contributed to the conditions necessary for sexual violence to occur, whether through silence or outright denial, misuse of authority, or harmful cultural norms. References to the church's complicity such has historical abuse cases (Denney et al., 2018; Keenan, 2013) and the *#MeToo* movement (Colwell and Johnson, 2020; Everhart, 2020), were common. Because of this, the participants that affiliate with an organized religion experience this conflict of values. These participants draw strength from their religious beliefs but also must contend with the fact that the institutions and members of their religion have perpetuated sexual violence, a crime that they have spent much of their professional career helping survivors recover from.

Another major way that some of the participants felt that religion could be a barrier involved survivor clients being pressured by religious leaders to forgive their attacker as a prerequisite for healing. Participants perceived this pressured forgiveness as coercive and psychologically damaging, noting that it often led to additional shame and emotional harm. In contrast, participants emphasized a trauma-informed approach to forgiveness, consistent with current best practices regarding forgiveness work in therapy (Davis et al., 2013; Worthington and Sandage, 2016), encouraging survivors' to make that choice on their own terms.

One participant shared a powerful counter-narrative in which she consulted with an Imam and the Imam was adamant that the survivor should not repent for the sexual abuse they experienced. This instance underscored the fact that religious leaders and institutions' approach to sexual violence is a complex phenomenon with many layers and conflicting accounts. Collectively, these findings highlight that while religious and/or spiritual beliefs and practices can offer support to many survivor clients and counselors, they can also be a source of harm, requiring critical reflection and contextual sensitivity in counseling practice.

## Implications

### Implications for counselors

Several implications emerged from our findings for counselors. The participants that shared stories of hurtful experiences they or their clients had had with religious institutions or individuals should be honored and validated because, indeed, many religious institutions have caused harm to individuals throughout history. This should be engaged with complexity, however, because religious institutions could serve both as a source of strength as well as a barrier for healing.

Some of the participants' negative feelings regarding rushed or pressured forgiveness came with several implications. It is important for counselors to examine their own perceptions and opinions regarding forgiveness for human perpetrated trauma such as sexual violence. Once the counselor has identified how they feel, it is important to not project those beliefs onto the client but to collaborate with them to decide in a way that is congruent with their values and goals. While there is evidence that forgiveness can bring about positive psychological changes and growth (Davis et al., 2013; Worthington and Sandage, 2016), the counselor should make clinical decisions based on their client's values and goals. However, if a survivor client is self-shaming because they feel that they should forgive because of their religious convictions but are not ready to do that yet, the counselor should normalize these feelings and be competent enough to engage in dialogue with the client about these religious themes (Association for Spiritual, Ethical, and Religious Values in Counseling, 2009).

While there have been many times when religious institutions have been a source of harm, the participants also showed how religion has helped both them and their survivor clients. Counselors should acknowledge the complexities and nuances that come with organized religion's role in both healing and harming. Additionally, counselors should also be culturally competent enough to be able to engage in dialogue about religion and spirituality, even if they don't identify as religious or spiritual themselves or if their client(s) identify with a religious affiliation that is different from their own (Association for Spiritual, Ethical, and Religious Values in Counseling, 2009). Counselors can thus help draw on clients' spiritual strengths as an asset in counseling or help them on their healing journey if they suffered spiritual harm at the hands of religious individuals or institutions.

Lastly, counselors can remember the importance of spiritual wellness as a component of self-care (Ohrt et al., 2019; Posluns and Gall, 2020). Self-care and professional wellness are ethical obligations of mental health professionals to provide quality care (American Counseling Association, 2014; American Psychological Association, 2017). The findings from this study highlight the importance of counselors maintaining their spiritual wellness to prevent burnout and to continue to be an asset to clients.

### Implications for counselor preparation

Multicultural competence is an important part of preparing counselors to be effective mental health professionals (Ratts et al., 2016) and spirituality is a part of it (Lu et al., 2020). To serve this end, counselor educators and supervisors should be aware of and incorporate content and dialogue regarding the *Competencies for Addressing Spiritual and Religious Issues in Counseling* (Association for Spiritual, Ethical, and Religious Values in Counseling, 2009) into their educational and supervisory practices. Creating counselors that can competently engage with the wide range of religious and spiritual beliefs of their clients will facilitate better clinical outcomes in diverse populations (Richards et al., 2009). Here, counselor educators and supervisors may also incorporate content related to faith development (Fowler and Dell, 2006) and changes that may occur over time in response to life changes and aversity. This may also better prepare counselor educators to help prevent burnout of counselors in training and newer counselors.

## Limitations and directions for future research

There were several limitations to this study. The data analysis involved arranging quotes from each participant into broader themes. While the participants were given the opportunity to review their respective transcripts for accuracy and make revisions, they were not involved in the final themes' construction. They might have arranged their thoughts and feelings into different categories or themes. While triangulation did occur thanks to the coding team, it is possible that we mistook some of the participants' meanings at times.

Another limitation is that participants came from a variety of mental health disciplines. Although all were helping professionals who practiced counseling and psychotherapy, there were differences in training and paradigm. Furthermore, the participants who practice in different countries would also have differences in training, adhere to different ethical codes, and may have different understandings of spirituality in psychotherapy in their respective cultural contexts. These differences and lack of uniformity could have impacted the participants' responses to the interview questions given their experiences were culturally and regionally bound. A third limitation is that there were more only two male-identified participants compared to nine female-identified. While this may be generally reflective of the gender makeup of the mental health counseling profession (Data USA, 2022), this gender identity diversity limitation may significantly limit transferability. Future research could seek to have a more equal distribution of gender identities.

Given these limitations, we offer several directions for future research. The first recommendation to expand on this study, future researchers should continue to include diverse participants of other religious or spiritual identities (i.e., Buddhist, Jewish, Sikh). While religious and spiritual diversity was a strength of this research, this is only one study. Additionally, researchers could conduct multiple studies that each focused on a different religious or spiritual affiliation. This study highlighted that there are meaningful similarities in experience across religions and spiritualties, there are also meaningful differences that could be explored further.

Additional research is needed that incorporates the spiritual development of mental health professionals with different religious or spiritual identities who work with different traumas and clients with differing worldviews than their own. The use of other qualitative methodologies is also recommended. Researchers could conduct a grounded theory study that builds and tests theory regarding spiritual development and indirect exposure to adversity.

## Conclusion

Change and development in one's faith and/or spirituality across the lifespan is a normal process that may sometimes be facilitated by the indirect exposure to clients' trauma. This study provided new insight into the experiences of faith development in a sample of religiously and/or spiritually diverse counselors who work with survivors of sexual violence. The participants in this study shed light on the ways their faith or spirituality changed as a result of their work with sexual violence survivors. Additionally, participants shared their perspectives on the ways religion can serve as a barrier in survivor healing. Study limitations such as a lack of training uniformity and uneven gender distribution were provided and directions for future research were provided.

## Data Availability

The raw data supporting the conclusions of this article will be made available by the authors, without undue reservation.

## Appendix A: Interview protocol questions

1. What discipline are you a part of in the helping profession? Counselor? Social Worker? Psychologist?
2. What kind of setting are you currently working in?
3. What kind of clinical work are you engaged in?
4. How would you describe your religious or spiritual identity?
5. How would you describe your work with sexual violence survivors?
6. How do you see your identity as _____ intersecting with your work with survivors of sexual violence?
7. How would you say that this work has impacted you emotionally?
8. How has your work with survivors of sexual assault impacted or changed your worldview?
9. a. You mentioned that you've experienced _______. Can you tell me more about any experiences you may have had in your work that you might consider to be secondary traumatic stress?
10. How do you see your religious or spiritual beliefs impacting the way you cope with what you have seen or heard in this work?
11. How do you see that your religious or spiritual beliefs or practices have changed as a result of this work?
12. How do you see any of your other identities, such as your race, sexual orientation, or gender factoring into these experiences you've described today?
13. Is there anything else that you'd like to share?
